# Mental Health of Mothers of Children with Neurodevelopmental and Genetic Disorders in Pakistan

**DOI:** 10.3390/bs12060161

**Published:** 2022-05-24

**Authors:** Michelle Jin Yee Neoh, Livia Airoldi, Zarah Arshad, Wasmiah Bin Eid, Gianluca Esposito, Dagmara Dimitriou

**Affiliations:** 1 Psychology Program, Nanyang Technological University, Singapore 639818, Singapore; michelle008@e.ntu.edu.sg; 2 Department of Psychology and Cognitive Science, University of Trento, 38028 Rovereto, Italy; livia.airoldi@studenti.unitn.it (L.A.); gianluca.esposito@unitn.it (G.E.); 3 Sleep Education and Research Laboratory, UCL Institute of Education, University College London, London WC1H 0AA, UK; zara.arshad.17@alumni.ucl.ac.uk (Z.A.); wasminah.bineid.14@alumni.ucl.ac.uk (W.B.E.)

**Keywords:** mental health, neurodevelopmental disorders, genetic disorders

## Abstract

This study examined maternal mental health in mothers of children with neurodevelopmental and genetic disorders in Pakistan; maternal education and affiliate stigma were also tested. It was hypothesised that mothers of children with neurodevelopmental and genetic disorders would experience high levels of depression, anxiety and affiliate stigma, and that these variables would be mediated by the level of maternal education. Seventy-five mothers of children with neurodevelopmental and genetic disorders were recruited from “Special Needs” schools in Lahore and Islamabad. The results showed that the majority of mothers were clinically depressed and a large majority of mothers were clinically anxious. The distribution of mothers with clinical anxiety was significantly different among mothers with different levels of education (*p* < 0.05). Furthermore, mothers with higher levels of education had less affiliate stigma. This result suggests that maternal education may be a protective factor of mental health in mothers of children with neurodevelopmental and genetic disorders. Limitations, implications and future research are discussed.

## 1. Introduction

A large multi-country research supported by the World Health Organisation (WHO) showed that mental health illnesses in developed countries are almost twice as likely to receive treatment as compared to developing countries [1]. According to World Bank data [2], Pakistan is one of the lowest performing countries on human development indicators in South Asia, especially in education and stunting. Children in low and middle-income countries have significant levels of unmet psychological needs and 10–20% of children are estimated to have mental health difficulties [3]. In Pakistan, 2.49% of the population has a disability and 14% of the disabled population has intellectual disabilities (IDs) [4]. Similar to child health, maternal mental health is also a topic of concern in Pakistan. Maternal wellbeing determines a child’s mental and physical health and this influence is suggested to start very early in development. Research suggests that maternal depression in developing countries is much higher than developed countries [5]. General statistics show that in Pakistan’s rural areas, 25–48% of women experience antenatal depression and 28–36% of women experience postnatal depression. Similarly, in Pakistan’s urban areas, 18–39% of women experience antenatal depression whereas up to 28% experience postnatal depression [5]. However, the amount of research examining maternal mental health in South-Asian countries is limited [3]. Despite the higher prevalence of postpartum common mental health disorders in low- and lower-middle-income countries compared to high-income countries, there is a lack of research on maternal mental health in these countries [6]. Depression and anxiety have also been examined in pregnant women in Pakistan by Ali et al. [7]. These authors show that the overall prevalence of either anxiety and/or depression in this population was 70%. Maternal depression is found to be prevalent in mothers of children with neurodevelopmental disorders or genetic disorders that affect the intellectual ability of the child. Similarly, research on maternal depression and anxiety in a population of children with neurodevelopmental disorders is mostly conducted in developed countries and only a few studies have looked at this topic in Pakistan and South Asia. Having a child with special needs may result in challenging parenting demands that place parents under higher stress. Most studies assessing parental stress in a clinical sample have been conducted in developing countries with “White” middle-class parents [8]. Cross-cultural studies showed that there are differences in the levels of stress experienced by parents with different cultural backgrounds and this could be due to different reasons [9,10]. Valicenti-McDermott and colleagues [8] looked at parenting stress in parents of children with autism and other developmental disorders in Hispanic and African-American parents, showing higher levels of parenting stress in parents born in the United States and/or of non-Hispanic origins compared to parents of Hispanic origins/immigrants. Possible determinants of maternal psychological wellbeing for mothers of children with ID have also been studied in Pakistan. Abbas, Feroz and Alyana [11] looked at how depression, anxiety and stress were moderated by familial factors in mothers of children with IDs (child age and presence and gender of other siblings). Highest levels of depression and stress were associated with having younger children with ID, while having a male and a female child apart from the child with ID, was correlated with the lowest levels of depression, anxiety and stress. These findings suggest that depression, anxiety and stress in mothers of children with ID can be moderated by child age and gender/number of siblings. Child mental health is consistently found to be associated with maternal health, even in lower-middle-income countries such as Pakistan [3]. Anxiety and depression separately and together were assessed in parents of children with ID in Pakistan. Azeem et al. [12] found that more than three quarters of parents had a clinically significant diagnosis of depression, anxiety or both. A significant association was found between a mother’s mental health and the degree of ID in their children; however, this association was not found among fathers. These results further suggest that in developing countries such as Pakistan, child disability impacts parents’ well-being significantly, and, similar to developed countries, it can be associated with components of parents’ mental health.

### 1.1. Maternal Education

The effect of maternal education on child health has been shown cross-culturally [13,14,15]. Lakshman et al. [16] examined indicators of child health and their associations with maternal education in the UK, China (rural areas) and Sweden. Children’s height was positively associated with maternal education, with the largest effect found in rural settings in China. Child stunting (malnutrition) was negatively associated with maternal education in the UK and in China, even though the latter had much higher stunting levels. In China, maternal education was positively associated with offspring’s weight and the prevalence of underweight children was highest in China as compared to the UK and Sweden. The overall BMI (body mass index) of children was inversely associated with maternal education in the UK and Sweden. These results suggest that maternal education is associated with one of the strongest predictors of child health and nutrition in not only developed countries but also developing ones, and weight, obesity, underweight and thinness of a child, in most cases, is also affected by maternal education. Similarly, maternal education is key in determining the child’s wellbeing in South Asia as well. Aslam and Kingdon [17] looked at the association between parental schooling, child health outcome and parental health-seeking behaviour in Pakistan. Their findings suggest that maternal education leads to long-term changes in height and weight, whereas paternal education leads to a change in short-term decisions related to child’s health (immunisation) in Pakistan. To summarise, maternal educational levels may affect later life choices made for the offspring.

### 1.2. Stigma and Affiliate Stigma Related to Child Disability in Developing Countries

Stigma is a negative attitude formed towards something without fair reason, which is often the case in mental health illnesses [18]. Such negative attitudes may lead to the formation of a negative mindset towards children and adults with mental health conditions, resulting in discrimination and exclusion [18]. The stigma related to mental health illness may affect not only the child, but also family members, such as mothers, who may become more worried not only for the present but also for the future of the child with a mental illness [19]. The inclusion and stigma related to a disability or illness varies across cultures, especially in developing countries. Patka and colleagues [18] examined the attitudes of community members and staff towards adults with ID. Being a staff member and having a friend or family member with ID was associated with a more positive attitude towards people with intellectual disability as compared to community members. Such findings reinforce the evidence about the negative attitude towards individuals with ID in Pakistan and low to middle-income countries. The presence of stigma towards individuals with neurodevelopmental and/or genetic disorders may impact parent’s opinions on their child’s disability as well. The attitudes of parents towards children with Down Syndrome (DS) have been investigated in Western countries as well as in a Pakistani sample. Ahmed et al. [19], looked at parental views on raising a child with DS in Pakistan and views on terminating pregnancies. Their findings, as well as others, show that the Pakistani society does not provide a supporting environment to raise special children, and there are limited health services for children with DS. Finally, lack of knowledge and education makes the understanding of intellectual and neurodevelopmental disorders more challenging [19]. The presence of stigma in the environment, may lead to the development of another form of stigma which is affiliate stigma (AS). AS is the internalisation of social stigma by family members and caretakers of individuals with different disabilities. Banga and Ghosh [20] looked at how AS impacts the wellbeing of mothers of children with specific learning disabilities (SLD) in India, showing a negative correlation between psychological well-being (Ryff Scale scores), affiliate stigma and subjective burden. A multivariate analysis showed that affiliate stigma predicted psychological well-being of mothers even after controlling for caregivers’ demographics and child characteristics. These findings suggest that AS predicts psychological wellbeing in mothers of children with SLD.

### 1.3. Aim of the Present Study

The aim of the present study is to assess the mental health (anxiety, depression) of mothers of children with neurodevelopmental and genetic disorders in Pakistan. Additional factors that may affect mental health, such as affiliate stigma and maternal education, are also investigated. We hypothesise that: (i) mothers of children with neurodevelopmental disorders or genetic disorders will have a clinically significant level of depressive and/or anxiety symptoms and (ii) maternal education will affect maternal mental health and affiliate stigma.

## 2. Methods

### 2.1. Participants

A total of 75 mothers (age: M = 37.1; SD = 6.2) of children (Age: M = 9.0; SD = 4.14; 44 males; 30 females; Birth Weight: M = 3.15; SD = 1.04) who attended “special education needs schools” were recruited from four schools in Lahore and Islamabad (capital of Pakistan). The schools were approached and invited to participate in the study in early January (2018). The schools then contacted the parents of children enrolled in the school. The schools in Islamabad invited parents by sending them the invitation letters beforehand. The schools in Lahore agreed to conduct data collection with parents visiting the school for either child-parent interactive sessions or with parents who came in for other services provided by the school (e.g., family support). Using a sample size calculator, the optimal sample size for this study can vary between 56–150 and we also considered other similar studies that were conducted previously in terms of determining the sample size (e.g., [11,12,19]). No specific developmental or genetic disorder in children was excluded, which means that no mothers were excluded based on the type of disorder their child had. The inclusion criteria were: (i) mothers of children with the following neurodevelopmental disorders—Cerebral palsy (6), intellectual disability (2), autism (13), developmental delay (1), ADHD (7), ADD (1), hyperactivity (4) and any other conditions that were not named previously (13) and (ii) mothers of children with genetic disorders such as Down Syndrome (12), microcephaly (4) and brain structure abnormalities including “smaller left hemisphere” or “bigger corpus callosum” (7).

### 2.2. Procedure

This study was approved by the Ethics Committee of the Institute of Education of University College London. Participants were recruited through the schools and asked to complete a questionnaire. Mothers were asked for an oral and written consent after the consent form was explained to them both in Urdu and English. Each participant was given time to understand the form and ask questions. The questionnaires participants completed were in English. Some women were not able to understand English so a volunteer helped with the translation. Participants were also told that they could ignore any question that would be stressful or that would affect them emotionally. Once the participants completed the questionnaire, they were thanked for their participation and debriefed.

### 2.3. Measures

#### 2.3.1. Demographic Questionnaire

Demographic information about the child and their mother was gathered using a questionnaire developed by the researchers. The questionnaire investigated different areas including maternal age, maternal education, child age, child gender, child weight at birth and child diagnosis (neurodevelopmental or genetic disorder). The level of maternal education was coded as 1 = individuals with a middle or high school education (8th to 12th class) and 2 = individuals with a tertiary education (B.A./B.Sc or M.A./M.Sc. equivalent). Seventy-three mothers provided information about their education.

#### 2.3.2. Depression and Anxiety Scales

Glasgow’s depression and anxiety scales were used to assess the levels of depression and anxiety in participants. These scales were chosen because of the simplicity of their questions (that include examples), the population targeted (expected to have some level of psychological abnormality) and psychometric strength. Cuthill, Epsie and Cooper’s adaptation (2003) of this self-report [21], for people with intellectual disabilities, was administered to participants. The scale consists of 20 questions assessing depression that were formulated after reviewing common depression scales and diagnostic criteria for depression. Answers to questions (regarding experiences in the last week) were gathered on a Likert scale of 1 (no) to 2 (a lot). A higher total would indicate a higher depression level, with a cut-off at 13 or above. For the assessment of anxiety, Mindham and Epsie’s [22] adaptation of Glasgow’s depression and anxiety scale was chosen because of its specificity for people with intellectual disabilities. The scale consists of 27 questions, assessing the following components of anxiety: worries (ten questions), specific fears (nine questions) and physiological symptoms (eight questions). Answers to questions regarding experiences in the last week were gathered on a 3-point Likert scale of 1 (no) to 2 (a lot). A higher total would indicate higher anxiety level, with a cut-off at 15 or above. Question 25 “Do you have to wee more often?” was changed to “Do you have to urinate/pee more often” as “wee” was not understood by the first participants.

#### 2.3.3. Affiliate Stigma Scale

The Affiliate Stigma Scale, designed by Mak and Cheung [23], was used to assess signs of AS in mothers of children with mental disorders. The word “person” was adapted to the word “child”, with the permission of the authors. In that way the questionnaire was made more specific to be used by mothers. For example, the question “I reduce going out with a person with mental illness/intellectual disability” was modified to “I reduce going out with a CHILD with mental illness/intellectual disability”. The word intellectual disability was not changed to neurodevelopmental or genetic disorder because, as noticed through the literature review, intellectual disability is a more common definition used to define types of mental impairments in Pakistani society. The AS Scale covers the cognitive, affective and behavioural components of AS with a total of 22 questions. Answers were recorded on a Likert scale of 1 (strongly disagree) to 4 (strongly agree). The scores in this questionnaire give an estimate for the level of AS experienced. The higher the score, the higher AS is experienced.

## 3. Analytic Plan

For Hypothesis 1, regarding the depressive and anxiety symptoms in mothers of children with neurodevelopmental or genetic disorders, we first calculated the descriptive statistics of the sample in terms of the percentage of mothers with clinical depression and clinical anxiety. A chi-square test was conducted to determine if the distribution of mothers with clinical anxiety and/or depression was significantly different. For Hypothesis 2 regarding the effect of maternal education on depressive and anxiety symptoms as well as affiliate stigma, we conducted chi-square tests to determine if maternal education and (i) clinical anxiety and (ii) clinical depression were independent. A *t*-test was conducted to determine if there were significant differences in stigma scores between mothers with middle/high school education and mothers with tertiary education. In order to examine the effect of maternal education on affiliate stigma while accounting for the relationship between depressive and anxiety symptoms and affiliate stigma, the following linear regression models were constructed: (i) anxiety symptom scores on affiliate stigma scores and (ii) depressive symptom scores on affiliate stigma scores. The residuals of these regression models were compared between mothers with middle/high school education versus mothers with tertiary education to evaluate the direction of the effect as described in [24].

## 4. Results

The descriptive statistics of the sample are summarised in Table 1. The results showed that 58.7% of mothers were clinically depressed (score of 13 and above) and 73.3% of mothers were clinically anxious (score of 15 and above). The mean score for depression was 13.88 (SD = 6.59); the mean score for anxiety was 20.04 (SD = 7.66).

For the analysis, mothers were grouped according to their depression and anxiety scores (Table 2). The mothers were grouped as follows: (i) mothers who were neither clinically depressed nor anxious (*n* = 13, 17.6%), (ii) mothers who were clinically anxious but not clinically depressed (*n* = 17, 23.0%), (iii) mothers who were clinically depressed but not clinically anxious (*n* = 6, 8.1%) and (iv) mothers who were both clinically anxious and depressed (*n* = 38, 51.4%). A significantly higher number of mothers in the sample were both clinically depressed and anxious, χ^2^ (1) = 8.24, *p* < 0.01, Cramer’s V = 0.33. Hence, the results support Hypothesis 1.

### 4.1. Anxiety and Maternal Education

A chi-square test found a significant difference in the distribution of mothers with clinical anxiety across the maternal education groups, χ^2^ (1) = 5.72, *p* = 0.03, Cramer’s V = 0.28 (Table 3). A greater proportion of mothers with middle/high school education had clinical anxiety (*n* = 28, 87.5%) compared to mothers with tertiary education (*n* = 25, 62.5%).

### 4.2. Depression and Maternal Education

No significant effect was found in the distribution of mothers with clinical depression across the maternal education groups, χ^2^ (1) = 0.41, *p* = 0.63 (Table 4).

### 4.3. Affiliate Stigma and Maternal Education

**Figure 1:**
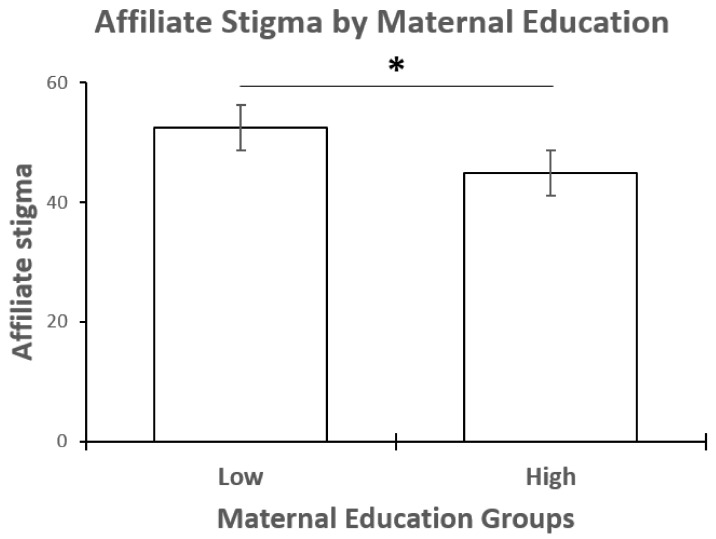
Group means of affiliate stigma scores by maternal education group. * = *p* = 0.001.

The mean score for affiliate stigma was 47.98 (SD = 9.82). An independent sample *t*-test found a significant difference in stigma score between the two groups of mothers, t(71) = 3.54, *p* = 0.001, and Cohen’s d = 0.83. Mothers with a middle/high school education had higher affiliate stigma scores (mean = 52.43; SD = 8.14) compared to mothers with a tertiary education (mean = 44.85, SD = 9.75) (Figure 1).

### 4.4. Affiliate Stigma and Maternal Education Excluding the Effect of Anxiety

A linear regression of anxiety scores on affiliate stigma scores was conducted and the standardised residuals were calculated. A lower mean of the residuals was observed in mothers with tertiary education (mean = −0.30; SD = 1.03) compared to mothers with middle/high school education (mean = 0.43; SD = 0.74).

### 4.5. Affiliate Stigma and Maternal Education Excluding the Effect of Depression

A linear regression of depression scores on affiliate stigma scores was conducted and the standardised residuals were calculated. A lower mean of the residuals was observed in mothers with tertiary education (mean = −0.30; SD = 1.03) compared to mothers with middle/high school education (mean = 0.43; SD = 0.74).

## 5. Discussion

The present study examined mental health and affiliate stigma in mothers of children with neurodevelopmental/genetic disorders in Pakistan, specifically in the cities of Lahore and Islamabad. The results showed that the majority of mothers were clinically depressed and a large majority of mothers were clinically anxious. The distribution of mothers with clinical anxiety was significantly different between mothers with different levels of education. This result suggests that maternal education may be a protective factor of mental health in mothers of children with neurodevelopmental and genetic disorders. This result aligns with previous studies that found a similar link between education and mental health disorders [25]. This finding has important implications due to the relationship between maternal education and child development outcomes. The importance of maternal education on child development outcomes has been well documented as parent educational level predicts knowledge of child care and child development [26]. Specifically, this result is consistent with existing literature investigating the role of maternal education on a number of key child development outcomes in low- and middle-income countries [27] including child mortality [13], efforts to support child learning and early child development outcomes [28]. A previous study also found that maternal education buffered the effect of maternal depression on children’s academic skills [29], suggesting that maternal education and maternal mental health interact in influencing child development outcomes. Hence, evidence supporting an association between maternal education and maternal mental health suggests that maternal education not only has repercussions on the mother, but also has far-reaching impacts on child development. Affiliate stigma was also significantly different between mothers of different education levels. Mothers with tertiary education reported experiencing lower levels of affiliate stigma compared to mothers with middle/high school education. The effect of maternal education on affiliate stigma remained significant even after controlling for the effect of anxiety and depression. This result suggests that maternal education may have an effect on how mothers perceive and respond to their child’s disorder and may be a key variable in reducing the experience of affiliate stigma. Additionally, mothers with higher levels of education may be able to better understand the disorder itself which could lower the possibility of feeling stigmatised due to their child’s disability. Specifically, higher maternal education may improve the understanding of a child’s health through the mechanisms of “Allocative Efficiency” and “Productive Efficiency” [30]. Allocative Efficiency implies that women with a higher education have greater knowledge and efficacy to combine health inputs in promoting infant and child health. Productive Efficiency is the ability to maximise the positive outcomes from a given set of positive inputs. An example of this could be being able to better communicate with doctors and understand their recommendations. Lastly, higher education may lead to the selection of a more educated partner, which in return, positively feeds back into the family, affecting the home environment, family decisions and socio-economic status. Therefore, it can be concluded that there are other factors that moderate psychopathology in mothers of children with disabilities in Pakistan, apart from education.

It is important to note that the present study was conducted in Pakistan and that there are likely to be cultural factors involved in the results observed in the present study. Although many studies have found evidence of poor mental health outcomes in mothers of children with special needs across different countries (see [31,32,33]), few studies have looked at the role of maternal education, especially in low-middle-income contexts where access to (higher) education may be limited. This suggests that mothers of children with special needs in Pakistan and other similar South Asian countries who do not receive a higher education may be a increased risk of psychopathology, relative to countries where higher education may be more accessible. There may also be different levels of stigma and different perceptions and experiences of this stigma in mothers of children with special needs in different countries (e.g., [34,35]). Hence, future studies can look to investigate maternal education and experiences of affiliate stigma across different cultures in order to elucidate cultural differences in the role of education and stigma on mental health outcomes of mothers with children with neurodevelopmental or genetic disorders.

### 5.1. Limitations

Apart from child behaviour, there are multiple variables that can affect maternal wellbeing, which might not be moderated by education. In a study looking at how maternal psychological wellbeing interventions affect mothers’ wellbeing in Pakistan, Zafar [36] reviewed multiple factors that affect maternal health. These factors included economic factors, sociodemographic factors (such as maternal age), social and family relationships, and domestic maltreatment.

The present study has some limitations, which include a small sample size and a broad range of child disabilities involved. The study was conducted in a low-middle-income country where recruitment of this particular population of mothers of children with neurodevelopmental or genetic disorders is difficult as well as constraints in recruitment of this population due to the sociocultural context that the study was conducted in. However, the aim of the present study was to explore possible associations between maternal education and the mental health of mothers of children with atypical development, rather than focusing on a specific group of children with particular pathologies. Hence, future studies can look to investigate the relationship between maternal education and mental health outcomes in mothers with children with particular types of pathologies, in order to more clearly distinguish between the mental health outcomes of caregiving for children with different types of neurodevelopmental and/or genetic disorders. As the present study focused on characterising the mental health profile of mothers with children with neurodevelopmental or genetic disorders, the mental health of mothers of children with typical development was not examined in this study. Future studies can recruit both samples of mothers of children with typical development and mothers of children with atypical development and compare the mental health of the two groups. Another big barrier was language. The national and first official language of Pakistan is Urdu, English is only the second official language. All tools, as well as consent and debrief forms, were in English. This means that the questionnaire may not have been as sensitive as a questionnaire in Urdu and there may be a bias in the sample as mothers with a higher educational level were more likely to be fluent in English. Finally, another limitation were the measures used to assess psychopathology in mothers. The measures used in this study, which are Glasgow’s Depression and Anxiety Scales, are designed to test depression and anxiety in a Western population sample. This means that it may not be suitable to assess depression and anxiety in Asian populations, where other cultural factors may come into play. Jayawickreme and colleagues [37] supports this view by comparing the effectiveness of 3 measures, of which one culture-specific, in predicting post-traumatic stress disorder (PTSD) in this sample. They showed that culture-specific tools are more sensitive to psychological distress, therefore demonstrating that Western tools may not always be the best measures for non-Western populations.

### 5.2. Implications

There are two main implications of the results of this research. Firstly, the results may be used to inform policies at the national level that target awareness not only of child disability but also of maternal mental wellbeing. Secondly, the results can be used to design workshops at local levels that target maternal wellbeing and promote positive societal attitudes as well as awareness of the medical causes of psychiatric disabilities in children. There is an urgent need to investigate the understudied situation of mental health in general in low- and middle-income countries with tools developed specifically for this purpose. The mental health of mothers is important as they are the primary caregivers of children with and without neurodevelopmental/genetic disorders. It would be valuable to conduct studies with a larger sample size, that integrate child variables as well as the paternal role in the upbringing of these children. Finally, the causal relationship between parent’s mental health and variables affecting mental health needs more research in low- to middle-income countries. 

## Figures and Tables

**Table 1:** Descriptive statistics of the sample.

	Mean	SD
Age	37.1	6.2
Depression score	13.88	6.59
Anxiety score	20.04	7.66
Affiliate stigma score	47.98	9.82

**Table 2:** Table of observed frequencies of clinical depression diagnosis groups by clinical anxiety diagnosis groups.

	Not Clinically Anxious	Clinically Anxious	Total
Not clinically depressed	13	17	30 (40.5%)
Clinically depressed	6	38	44 (59.5%)
Total	19 (25.7%)	55 (74.3%)	74

**Table 3:** Table of observed frequencies of maternal education groups by clinical anxiety diagnosis groups.

	Not Clinically Anxious	Clinically Anxious	Total
Middle/high school education	4	28	32 (44.4%)
Tertiary education	15	25	40 (55.6%)
Total	19 (26.4%)	53 (73.6%)	72

**Table 4:** Table of observed frequencies of maternal education groups by clinical depression diagnosis group.

	Not Clinically Depressed	Clinically Deppressed	Total
Middle/high school education	12	20	32 (44.4%)
Tertiary education	18	25	40 (55.6%)
Total	30 (41.7%)	42 (58.3%)	72

## Data Availability

Data are not publicly accessible but can be made available on request to D.D.
